# Resistance of Pastes from Carbonated, Low-Lime Calcium Silica Cements to External Sulfate Attack †

**DOI:** 10.3390/ma16124276

**Published:** 2023-06-09

**Authors:** Raikhan Tokpatayeva, Jan Olek, Sadananda Sahu

**Affiliations:** 1 Lyles School of Civil Engineering, Purdue University, West Lafayette, IN 47907, USA; olek@purdue.edu; 2 Solidia Technologies Inc., Piscataway, NJ 08854, USA; sahu2u@gmail.com

**Keywords:** carbonation, calcium silicates, sulfate resistance

## Abstract

This paper presents the results of a study on the evaluation of resistance of pastes from carbonated, low-lime calcium silica cements to external sulfate attack. The extent of chemical interaction between sulfate solutions and paste powders was assessed by quantifying the amount of species that leached out from carbonated pastes using ICP-OES and IC techniques. In addition, the loss of carbonates from the carbonated pastes exposed to sulfate solutions and the corresponding amounts of gypsum formed were also monitored by using the TGA and QXRD techniques. The changes in the structure of silica gels were evaluated using FTIR analysis. The results of this study revealed that the level of resistance of carbonated, low-lime calcium silicates to external sulfate attack was affected by the degree of crystallinity of calcium carbonate, the type of calcium silicate, and the type of cation present in the sulfate solution.

## 1. Introduction

Growing interest in reducing CO_2_ emissions associated with production of ordinary Portland cement (OPC) has spurred efforts to develop alternative, more “eco-friendly” types of binders. Examples of these types of binders includes cements composed chiefly of low-lime calcium silicates, such as wollastonite/pseudo-wollastonite (CaO·SiO_2_) and rankinite (3CaO·2SiO_2_). While the production of such cements requires lower amounts of limestone, and hence offers the potential to reduce CO_2_ output, these materials are non-hydraulic. However, when exposed to liquid water, these cements will react with CO_2_. That reaction results in a hardened product, consisting of calcite and silicate phases, which serves as a binding medium in production of mortars and concretes, thus offering another opportunity to effectively reduce CO_2_ output by permanently sequestering it in the reaction product.

In general, the published literature contains a very limited number of papers focusing on the durability of carbonated (low-lime) calcium silicates (CCS). According to the work published by Tokpatayeva et al. [1] and Jain et al. [2], when exposed to both sodium and magnesium sulfate solutions, the CCS mortar bars tested as per ASTM C1012 showed significantly reduced expansion (<<0.1%) compared to OPC ones. Nevertheless, according to R. Tokpatayeva et al. [3], the CCS system was somewhat reactive when exposed to MgSO_4_, but it was not reactive when exposed to Na_2_SO_4_. Specifically, these authors reported that despite the absence of portlandite and reactive aluminates in the matrixes of the CCS mortar bars (made with Solidia^TM^ cement), prolonged exposure to magnesium sulfate resulted in microcracking, decalcification of the matrix, and formation of gypsum. Moreover, traces of magnesium species were found in the amorphous silica phase. Finally, data presented in [3] indicate improved sulfate resistance of the CO_2_-cured OPC-based matrix.

The study conducted by Quin et al. [4] suggested that unlike in the case of OPC materials, the low temperature exposure of CCS matrixes to external sulfate attack does not result in formation of the thaumasite. Specifically, these authors concluded that, at low temperatures, the sulfate-rich environment causes decalcification of the matrix and precipitation of gypsum crystals. Similarly, the study by Zhang et al. [5] reported that no thaumasite formed at low temperature in carbonation-cured OPC and Portland-limestone cement (PLC) specimens. Moreover, the carbonation curing in both matrixes improved the sulfate resistance at low temperature. To return to the studies described in [4], it is worthwhile to note that the authors revealed a certain dependence of the behavior of the CCS on the type of the sulfate cation as well as the type of the calcium silicates. However, given possible variations in the type of sulfate cations and calcium silicate minerals involved in the reactions, a more in-depth investigation (in terms of chemical interaction of the CCS phases with sulfate species) is warranted.

The goal of this study was to evaluate the resistance of pastes made from carbonated, low-lime calcium silica (CS) cements to external sulfate attack by exposing them to three types of sulfate solutions: sodium sulfate, magnesium sulfate, and aluminum sulfate. The low-lime CS cements used for preparation of the pastes included wollastonite (cement #1), amorphous CS (cement #2), and two types of commercially produced low-lime binders (cements #3 and #4) (SolidiaTM cements). The sulfate attack environment was simulated by storing slurries (made from pulverized carbonated cement pastes mixed with sulfate solutions) at room temperature (lab environment) for periods of up to 120 days. The stored slurries were periodically sampled (at the age of 0.5, 1, 5, 10, 20, 60, 90, and 120 days) and centrifuged (to separate solids from liquids). The solid phase was analyzed using TGA, XRD, and FTIR techniques, while the liquid phases were analyzed by ion chromatography (IC) for the sulfate species, and by inductively coupled plasma optical emission spectroscopy (ICP-OES) techniques for all other species, including calcium, silica, alumina, magnesium, and sodium.

## 2. Description of the Analytical Methods

### 2.1. Thermogravimetric Analysis (TGA)

The TGA of paste samples was performed using the Q50 apparatus from TA Instruments. In preparation for the TGA tests, the hardened paste specimens were first pulverized (using mortar and pestle), and the resulting powder was then passed through a 75 μm (#200) sieve. Next, about 15–20 mg of the screened powder was placed in an open platinum pan which was then loaded into the TGA instrument. The pan with powder was heated to 900 °C (at a rate of 10 °C/min) in the atmosphere of nitrogen gas. The flow rate of the gas was 60 mL/min.

### 2.2. X-ray Diffraction (XRD) Analysis

The XRD analysis was performed using a Siemens D500 diffractometer with CuKα radiation operated at 50 kV and 30 mA. The analysis was performed using powdered samples mounted in an aluminum holder. The samples were scanned over the 2θ range of 5–60° at a rate of 0.02° 2θ/s. The identification of the diffraction peaks and the Rietveld analysis were performed with the aid of Jade 9 and Profex 4.3.5 software packages, respectively.

### 2.3. Fourier Transform Infrared (FTIR) Analysis

FTIR spectra of the powder samples were acquired over the range of 600–3000 cm^−1^ using the Perkin Elmer Frontier FTIR Spectrometer (Perkin Elmer, Waltham, MA, USA). For every spectrum, an average total of 10 scans were recorded with a resolution of 4 cm^−1^.

### 2.4. Soak Solution Analysis

The determination of concentrations of ionic species present in the filtered soak solutions was performed using inductively coupled plasma optical emission spectroscopy (ICP-OES) and ion chromatography (IC) techniques. The Thermo Scientific iCAP 7400 spectrometer (Perkin Elmer, Waltham, MA, USA) was used for the ICP-OES analysis, while IC testing was performed using the DIONEX ICS-900 (Sunnyvale, CA, USA) instrument. Before testing, the instruments were calibrated using the standard solutions from VWR Chemicals BDH^®^. The test samples for the ICP-OES test were acidified using 2% (by volume of the specimen) nitric acid.

## 3. Results of Characterization of Cements and Reference Pastes

### 3.1. Low-Lime Calcium Silicate Cements

**Figure 1:**
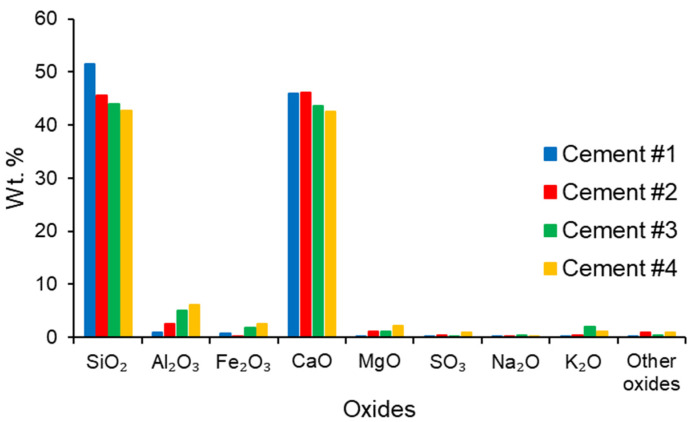
Oxide compositions (%) of the low-lime calcium silicate cements.

Four different types of low-lime calcium silicate cements, labeled #1, #2, #3, and #4, were used to prepare paste specimens used in this study. These cements were characterized by both the X-ray fluorescence (XRF) and X-ray diffraction (XRD) techniques to elucidate, respectively, their oxide and mineralogical compositions. The oxide compositions of the cements are shown in Figure 1.

Based on the data presented in Figure 1, it can be seen that while the CaO contents of all cements were very comparable (43–46%), their SiO_2_ contents varied from 51.5% (for cement #1) to 42.7% (for cement #4). In addition to these two main oxides, all cements also contained variable amounts of other (minor) oxides, including Al_2_O_3_, Fe_2_O_3_, MgO, and K_2_O. These oxides were present in the lowest quantities in cement #1 and in the highest quantities in cement #4. The values of the loss on ignition (LOI) and the XRF-derived amounts of total alkalis present in these cements are given in Table S1. The values of the LOI of cements #1, #3, and #4 were very comparable, whereas the value of the LOI of cement #2 was about twice as high as that of the other cements. The total alkalis content was lowest in cement #1, whereas it was highest in cement #3.

The XRD analysis showed significant diversity in the crystallographic makeup of the cements (see Figure S1). Cement #1 consists of almost 100% natural wollastonite while cement #2 appears to be composed of mostly amorphous calcium silicate, as indicated by the presence of a broad hump in the two-theta range of ≈26–34°. Cements #3 and #4 both contain a mixture of several crystalline phases, and their mineralogical compositions are very similar. The majority of the XRD pattern peaks present in these two cements belong to larnite (β-2CaO·SiO_2_), pseudo-wollastonite (CaO·SiO_2_), rankinite (3CaO·2SiO_2_), gehlenite (2CaO·Al_2_O_3_·SiO_2_), sanidine feldspar ((K, Na) (AlSi)_4_O_8_), quartz (SiO_2_), and cristobalite (SiO_2_). The main differences in the mineralogical compositions of cements #3 and #4 are related to the relative proportions of the minerals present. Specifically, cement #3 contains more calcium silicates (larnite, rankinite, and pseudo-wollastonite) than cement #4. In contrast, cement #4 contains more gehlenite and sanidine feldspar. Moreover, the narrow peaks visible in the XRD pattern of cement #4 indicate the presence of a well-developed crystalline phases. On the other hand, peaks present in cement #3 are broader and less defined, implying a lower degree of crystallinity.

### 3.2. Carbonated Reference Pastes

#### 3.2.1. Preparation of the Pastes

All paste samples were prepared by hand-mixing of small amounts of cements with deionized water (w/c = 0.35) and placing a thin (1.0–1.5 mm thick) layer of the resulting slurry in VWR sterile polystyrene Petri dishes (ϕ = 60 mm and h = 15 mm). Immediately after finishing the placing of the slurry, the paste-containing Petri dishes were transferred to a VWR Symphony 1.4A CO_2_ incubator where they were carbonated for a period of 5 days. The carbonation was carried out in a 20% carbon dioxide environment at the temperature of 23 °C and relative humidity of 90–100%. Once carbonated, the paste samples were pulverized (using mortar and pestle) and stored in sealed glass vials until needed for further testing. Before being exposed to sulfate solutions, the powdered carbonated paste samples were characterized using TGA, XRD, and FTIR techniques.

#### 3.2.2. Results of the Thermogravimetric Analysis (TGA) of the Pastes

The amounts of calcium carbonates present in carbonated pastes were estimated from the TGA thermograms by analyzing weight losses occurring in two temperature ranges: 650–800 °C and 450–630 °C. The weight loss observed in the temperature range 650–800 °C is the result of decomposition of highly crystalline calcium carbonate phase–mainly calcite. On the other hand, weight losses observed within the lower temperature range are associated with decomposition of less crystalline forms of calcium carbonate, namely, vaterite. The results of these quantitative examinations are shown in Table 1. Analysis of the first derivative of the TGA curves (i.e., the DTGA) in the temperature range 450–630 °C, presented in Figure S2, reveals the presence of the small hump in data obtained from pastes made with cements #2, #3, and #4.

#### 3.2.3. Results of the X-ray Diffraction (XRF) Analysis of the Pastes

The XRD patterns of paste samples are presented in Figures S3 and S4. It can be seen that all carbonated paste samples contain calcite. However, peaks of vaterite were also observed in the pastes made from cements #2, #3, and #4. The vaterite peaks are relatively easily distinguishable in pastes from cements #3 and #4 paste samples. However, local magnification of the relevant segment of the thermogram was needed to reveal the vaterite peaks in the XRD pattern of the cement #2 paste (see Figure S3b).

#### 3.2.4. Results of the FTIR Analysis of the Pastes

**Figure 2:**
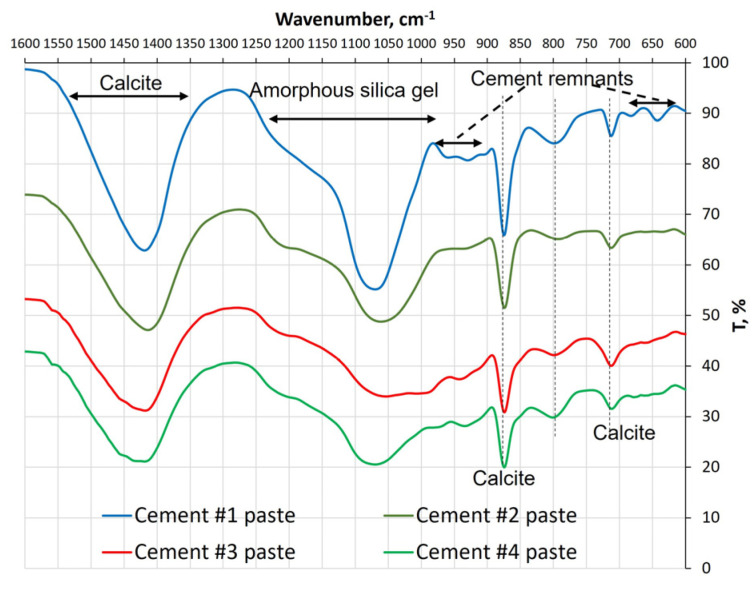
FTIR spectra of the carbonated reference (i.e., not exposed to sulfate solutions) paste samples.

The FTIR absorption spectra of the carbonated reference (i.e., not exposed to sulfate solutions) pastes are shown in Figure 2. The bands with the peaks at around 1420, 874, and 712 cm^−1^ belong to calcite and are visible in spectra of all four pastes. According to Milkey [6], who examined the FTIR spectra of different feldspar minerals, the sharp and well-defined peaks indicate the presence of a mineral with a well-developed crystalline structure. Applying this reasoning to data presented in Figure 2, it can be concluded that the calcite formed in the cement #1 paste is more crystalline than that present in other paste samples. Regarding other polymorphs of calcium carbonate, the aragonite spectrum is similar to that of calcite except for the presence of two doublets in the wavenumber ranges of 850–900 cm^−1^ and 670–714 cm^−1^ [7,8]. No such doublets were observed in any of the spectra shown in Figure 2. This observation confirms the results of the XRD analysis, which also did not reveal the presence of the aragonite. On the other hand, it was challenging to detect the presence of the vaterite in the FTIR spectra even though this mineral was visible in XRD patterns of all pastes except the one produced from carbonated wollastonite (i.e., cement #1). This is because the FTIR peaks characteristic of vaterite (at wavenumbers ~1070 and 873 cm^−1^ [9,10]) overlapped with those assigned to silica and calcite, respectively. The FTIR curves did not contain a band located at ~745 cm^−1^ which is typically interpreted to represent absorption due to deformation of carbonate ion. However, the band was located at ~848 cm^−1^ which appeared as a shoulder on the calcite band (~874 cm^−1^) in the spectra of cements #3 and #4 paste samples.

The strong broad band located between 1000 and 1300 cm^−1^ and a smaller one located at ~800 cm^−1^ likely belongs to amorphous silica [9,11,12]. The former band, according to the published data [6,7,13,14,15], occurs due to Si-O stretching vibrations in all silicate structures. However, our data indicate that the shape and intensity of that band vary, depending on the type of the cement used to prepare the paste. Launer [16] points out that the Si-O band tends to shift towards the higher wavenumber values and becomes narrower as Si/O ratio increases. Furthermore, as stated by Yu et al. [17], in CSH-like gels (C/S ≤ 1.2), the shift of the Si-O band in the direction of lower wavenumbers is mainly associated with the depolymerization of silicate chains and the increase in the Ca/Si ratio. Based on these reported findings, one can conclude that the amorphous silica gel present in the carbonated pastes prepared from cements #2, #3, and #4 are likely modified with calcium ions.

## 4. Sulfate Exposure Experiment

### 4.1. Experimental Setup

The concentration of all sulfate solutions used in this study was 0.35 M. The solutions were prepared using ACS reagent-grade chemicals (anhydrous sodium sulfate, anhydrous magnesium sulfate, or 18-hydrate aluminum sulfate) and deionized water. The pH values of the sulfate solutions prior to contact with the paste powders were in the range of 6–7 (for sodium and magnesium sulfate types), and in the range of 3–4 for the aluminum sulfate type.

The test specimens consisted of slurries prepared by mixing paste powders with sulfate solutions (at solid-to-liquid ratio of 1:4 by volume) in plastic centrifuge tubes with 50 mL capacity. Once filled with slurries, the centrifuge tubes were sealed and stored (cured) in an ambient lab environment (23 ± 5 °C). While being stored, the tubes were manually agitated twice a week. While being mixed with aluminum sulfate solution, all paste powders released CO_2_ gas bubbles (the result of the decomposition of calcium carbonates). Therefore, these slurries were sealed only after the bubbling of CO_2_ had ceased. For comparison purposes, the paste slurries were also prepared by mixing CS with deionized water (these will be referred to as control specimens). The total duration of the sulfate exposure test was 120 days. After 0.5, 1, 5, 10, 20, 60, 90, and 120 days of exposure, a total of 16 samples (4 different paste slurries per each type of solution, including the control) were removed from the storage vials and used for post-exposure analysis.

### 4.2. Post-Exposure Analysis

At the end of each of previously mention period of exposure, the slurries were placed into the centrifuge lined with 0.2 µm cellulose acetate filter to separate their solid and liquid components. After separation, the solids were oven-dried (at 35 ± 5 °C) for three days, ground (using mortar and pestle), and placed in sealed glass tubes where they were stored until test time. These solids were analyzed using the TGA, XRD, and FTIR methods. The extracted liquids (soak solutions) were transferred to 15 mL plastic centrifuge tubes and stored in the laboratory refrigerator at ~4–5 °C until they were analyzed by ICP-OES and IC techniques.

## 5. Results of Sulfate Exposure Experiments

### 5.1. Soak Solution Chemistry Results

**Figure 3:**
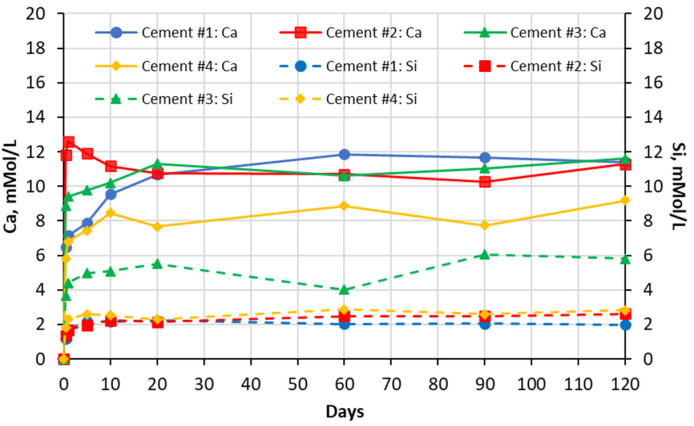
Changes in concentrations with time of Ca and Si species present in sodium sulfate solutions in contact with cement paste specimens.

**Figure 4:**
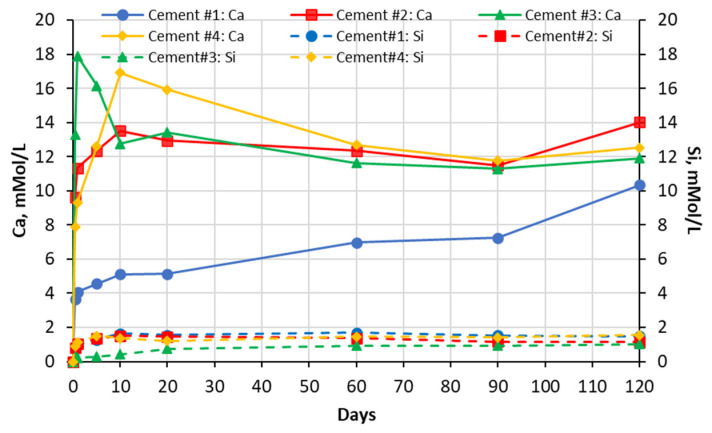
Changes in concentrations with time of Ca and Si species present in magnesium sulfate solutions in contact with cement paste specimens.

**Figure 5:**
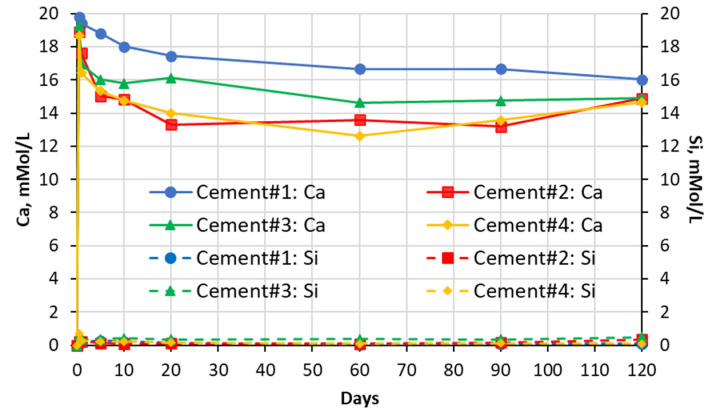
Changes in concentrations with time of Ca and Si species present in aluminum sulfate solutions in contact with cement paste specimens.

**Figure 6:**
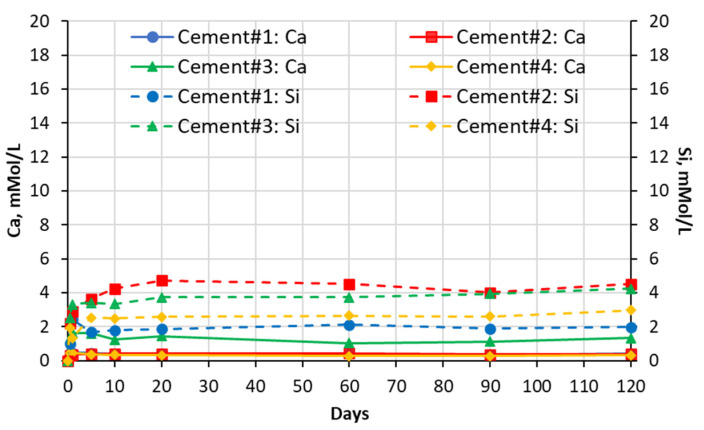
Changes in concentrations with time of Ca and Si species present in deionized water (DIW) in contact with cement paste specimens.

The results of the chemical analysis of soak solutions showed that the sulfate-rich environment significantly influences the leaching behavior of calcium and silica ions from the carbonated calcium silicate (CCS) matrixes. As shown in the graphs presented in Figure 3, Figure 4, Figure 5 and Figure 6 exposure to all sulfate solutions resulted in a significant (10–15 times) increase (compared to deionized water) in the amount of calcium ions leached out from all CCS matrixes. Another noticeable trend was a gradual decay (over time) in the concentration of the Ca ions present in aluminum sulfate soak solutions in contact will all four types of CCS pastes. The same trends were also observed in the case of sodium sulfate soak solution in contact with cement #2 paste and in the case of magnesium sulfate soak solutions in contact with pastes prepared with cements #3 and #4. These trends can be explained by the formation and precipitation of new compounds such as gypsum (see data presented in Section 5.2 and 5.3 of this paper).

The effect of sulfate solutions, especially aluminum and magnesium sulfates, on the leaching of silica species appears to be quite opposite to that observed for calcium ions. As an example, when compared to the case of exposure to deionized water, only negligibly small amounts of silica were found in aluminum sulfate soak solutions in contact with any of the four CCS pastes. This implies that aluminum species may be stabilizing silica species present in the CCS. Similar behavior was observed in the case of CCS exposed to magnesium sulfate solutions, in that magnesium appears to be stabilizing (although somewhat less effectively than aluminum) silica in all CCS paste samples. Finally, the effect of sodium sulfate solution appears to be somewhat mixed as (when compared to the effect of deionized water) it reduced the leaching of silica in cement #2 paste samples, but it increased it cement #3 samples.

Both, the initial (i.e., before they came in contact with the paste powders) and final (i.e., determined at the end of the 120-day exposure period) pH values of the deionized water and the three sulfate solutions are shown in Figure S5 in the Supplementary Materials section of the paper.

The initial pH of the deionized water was ~7, and these values were in the range of 6–7 for sodium and magnesium sulfate solutions. On the other hand, the aluminum sulfate solution was rather acidic, with pH levels in the range between 3 and 4. At the end of the 120-day exposure period, the pH values of the soak solutions reached the following values:

- Sodium sulfate: 8–9 for cements #1, #2, and #4 paste samples and 10 for cement #3 paste sample.
- Magnesium sulfate: ~8 for all cement paste samples.
- Aluminum sulfate: ~7.5–7.8 for all cement paste samples.
- Deionized water: ~9–10 for all cement pastes.

As previously reported in [18], the pH of the aqueous media significantly affects the solubility of calcium carbonate. The initial pH values of all sulfate solutions (before addition of the CCS paste powders) were low enough to destabilize the CaCO_3_ phases, as confirmed by the increased concentration of calcium species present in sulfate soak solutions compared to the concentrations present in the deionized water.

**Figure 7:**
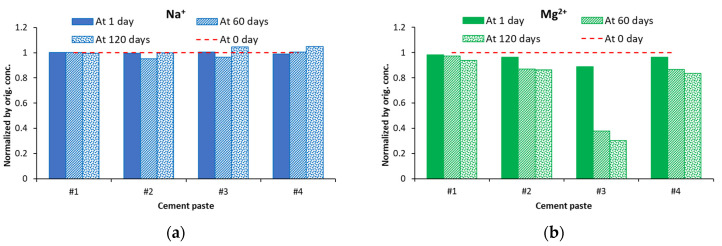
Changes in normalized concentrations of (**a**) Na^+^ and (**b**) Mg^2+^ ions in soak solutions of all cement paste samples after selected periods of exposure.

The changes in concentrations of sodium and magnesium ions after 1, 60, and 120 days of exposure (normalized with respect to the concentration at 0 days) are presented in Figure 7. Due to the negligible low values of the concentrations of aluminum cation (and the associated sulfate anion), these results are not presented. As shown in the graphs presented in Figure 7a, concentrations of sodium ions stay almost constant, with the exception of a slight reduction in cases of the cements #2 and #3 pastes at 60 days and a slight increase in cements #3 and #4 at 120 days. However, as can be seen from Figure 7b, at 120 days, the concentration of magnesium ions was reduced by as much as ~68% for the cement #3 paste sample and by ~10% in the cases of cements #2 and #4 paste samples. After 120 days of exposure, the reduction in concentration of magnesium ions in soak solution in contact with cement #1 paste samples was very small—only ~2%.

**Figure 8:**
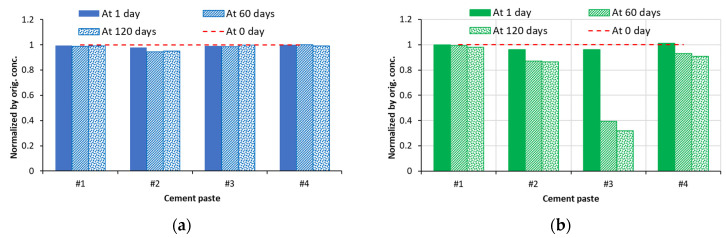
Changes in normalized concentrations of sulfate ions in (**a**) sodium sulfate and (**b**) magnesium sulfate soak solutions of all cement paste samples after selected periods of exposure.

The changes in concentrations of sulfate ions after 1, 60, and 120 days of exposure to sodium and magnesium soak solutions (normalized with respect to the concentration at 0 days) are presented in Figure 8. As seen from the graphs presented in Figure 8, the existing trends seem to be quite similar to those observed for the corresponding sodium and magnesium cations.

### 5.2. XRD Analysis Results

**Figure 9:**
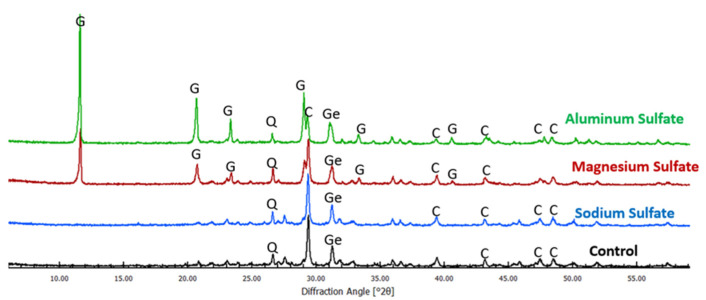
XRD patterns of the cement #3 paste samples after 120 days of exposure to sodium, magnesium, and aluminum sulfate solutions (key: C—calcite, G—gypsum, Ge—gehlenite, Q—quartz).

The analysis of the XRD patterns indicates that the degree of alteration of the existing crystalline phases as well as the formation of the new crystalline phases depend on both, the type of paste and the type of sulfate solution. In general, the biggest observed change resulting from the exposure of pastes to various sulfate solutions was the formation of gypsum (mostly at the expense of calcite) in all pastes exposed to aluminum sulfate. In addition, formation of gypsum was also observed in pastes #2, #3, and #4 exposed to magnesium sulfate as well as paste #2 exposed to sodium sulfate. For results obtained for paste #3 see Figure 9; the XRD curves for the rest of the paste samples are given in Supplementary Materials (Figures S6–S8).

**Figure 10:**
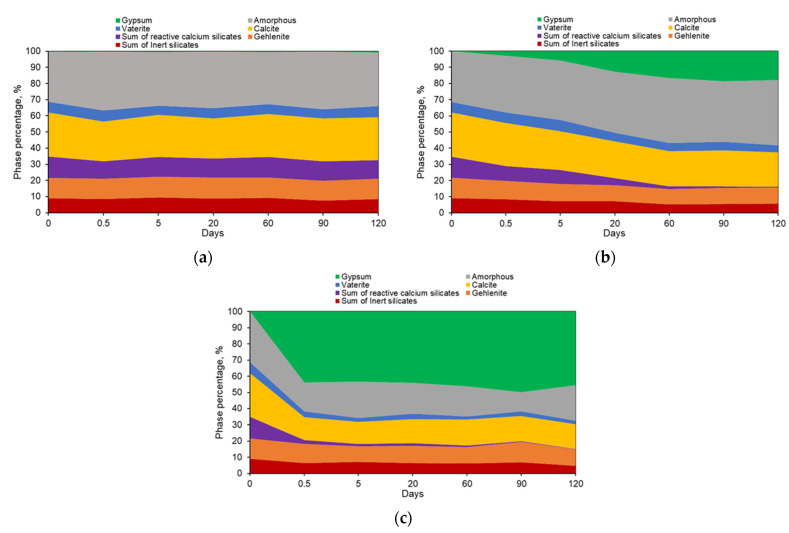
The results of the QXRD analysis of cement #3 paste samples after exposure to (**a**) sodium, (**b**) magnesium, and (**c**) aluminum sulfate solutions.

To quantify the amounts of crystalline and amorphous phases present in the pastes during exposure to sulfates, the Rietveld refinement procedure was implemented, and the content of calcite (previously independently determined via the TGA method) was utilized as an “internal” standard. The results of the phase quantification procedure for each type of cement paste are presented as cumulative diagrams in Figures S9–S11. Here, as an example, the QXRD analysis results of cement #3 paste are presented in Figure 10. No significant changes could be observed in the paste samples submerged in sodium sulfate solution (except for the formation of an almost negligibly small amount of gypsum) (see Figure 10a). Due to gypsum being present in such a small amount, it is hard to identify exactly which phase was involved in its formation. However, as it can be seen in Figure 10b that the exposure to magnesium sulfate solution resulted in noticeable changes with respect to types and amounts of the phases. First, a significant portion of the mass of the sample was associated with gypsum, the amount of which increased with the increase in the length of the exposure period. In addition, the increase in the amount of the amorphous phase can also be observed. All these increases seem to be counterbalanced by the decrease in the amounts of calcium carbonates and reactive calcium silicates. Furthermore, by the end of the 120-day exposure period, a slight reduction in the amount of gehlenite was also observed. Submersion of the cement #3 paste powder samples in aluminum sulfate solution resulted in the production of a large amount of gypsum (~40–45%) shortly after the test started (after approximately a half day of exposure), decomposition of almost half of the amount of calcium carbonates, and the almost complete depletion of the reactive calcium silicates (see Figure 10c).

Regarding other paste samples, as shown in Figure S9a,b, the carbonated wollastonite paste (cement #1) does not seem to have experienced any change in composition when exposed to either sodium or magnesium sulfate solutions. However, as can be seen from the graph in Figure S9c, at the end of the 120-day exposure period, there is about a 60% decrease in the amount of calcite and about a 40% decrease in the amount of wollastonite when this paste was exposed to aluminum sulfate. The amount of gypsum seems to remain more or less constant after the first half day of exposure and decreases only slightly by the end of the test.

Unlike cement #1 paste samples, the cement #2 paste samples were observed to undergo compositional changes upon exposure to all three types of sulfate solutions (see Figure S10). In the case of both sodium and magnesium sulfates, almost similar amounts of gypsum appeared in the system. Regardless of the sulfate solution used, no noticeable change in the amount of the amorphous phase can be detected. Similar to cement #1 paste, there was a drastic compositional change in samples exposed to aluminum sulfate solution. First, the reduction of calcite amount and complete disappearance of vaterite took place. The amount of gypsum (formed almost immediately, i.e., within the first 0.5 day of exposure) seems to stay almost constant throughout the entire exposure period.

The QXRD results of the cement #4 paste samples after exposure to sodium sulfate solution did not reveal any significant changes in the amount or in the type of phases, as illustrated in Figure S11a. On the other hand, changes were observed in samples immersed in magnesium sulfate solution. Specifically, gypsum was observed to form, the amount of which increased with the increase in the time of exposure. This was accompanied by the decrease in the amount of calcium carbonate phases and reactive calcium silicates (see Figure S11b). The trend of changes occurring in samples exposed to aluminum sulfate, presented in Figure S11c, looks similar to what was observed in the case of cement #3 paste samples.

### 5.3. Thermal Analysis Results

**Figure 11:**
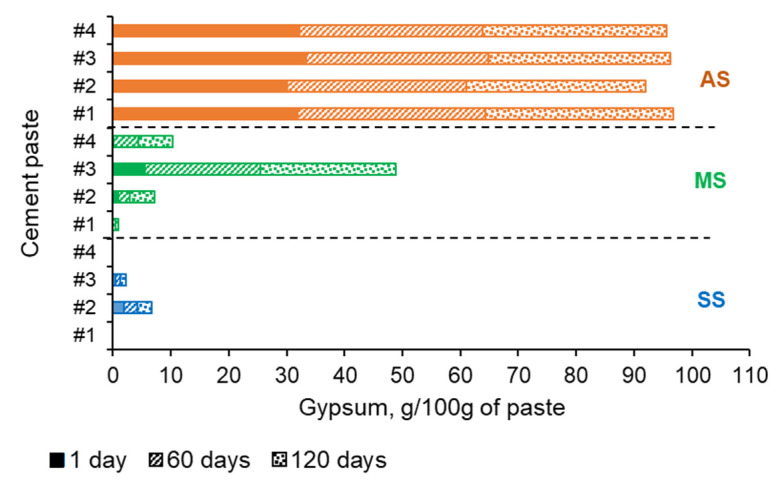
Amount of gypsum formed during exposure to SS—sodium, MS—magnesium, and AS—aluminum sulfate solutions.

The thermal analysis (TGA) of the paste samples after certain periods of exposure was used to quantify any changes in the amount of calcium carbonate (mainly calcite) and amount of gypsum formed during the sulfate exposure period. In order to eliminate the overestimation of the gypsum quantities resulting from the possible interference of continuous dehydration of the amorphous silica gel in the temperature range of ~90–150 °C, the methodology described by Kim and Olek [19], originally developed for the estimation of calcium hydroxide, was implemented. The values of the gypsum amount formed after 1, 60, and 120 days of exposure are plotted in the graphs shown in Figure 11.

Exposure to sodium sulfate solution did not cause formation of significant amounts of gypsum compared to other sulfate solutions (see Figure 11). Only a small amount (about 2.5 g of gypsum per 100 g of paste) was detected in cement #2 paste samples at the end of the exposure test. Opposite to that, magnesium sulfate solution triggered the precipitation of much more significant amounts of gypsum in all paste samples except in the case of cement #1 pastes (carbonated wollastonite) (only about 0.5 g/100 g of paste formed). Exposure to aluminum sulfate solution resulted in the formation of about 30–32% (wt. of paste) of gypsum in all pastes within the first 12 h of testing (Figure 11). This can be explained by the high acidity of the solution (pH ≈ 3–4) which resulted in the vigorous decomposition (“digestion”) of the calcium carbonate phase, boosting of Ca ion release to the solution, pH stabilization at the level of ~6–7, binding of released calcium ions by sulfate ions, and subsequent quick precipitation of gypsum which remained more or less stable throughout the entire exposure period.

The change in the amount of calcium carbonate (mainly calcite), normalized with respect to the amount of calcium carbonate present in carbonated pastes before the initiation of exposure to different sulfate solutions is illustrated in the graphs presented in Figure S12. Overall, the trends shown in Figure S11 seem to be somewhat correlated with the trends of gypsum, especially in the cases of cements #2–#4 paste samples submerged in magnesium sulfate solution (see Figure S12).

To verify the assumption that all the calcium carbonates lost during exposure to magnesium sulfate were consumed during the formation of gypsum, the comparison of the stoichiometric amount of calcium carbonate needed for the formation of gypsum and the amount of calcium carbonate lost during the experiment was performed. The details are given in Appendix A.

### 5.4. FTIR Analysis Results

**Figure 12:**
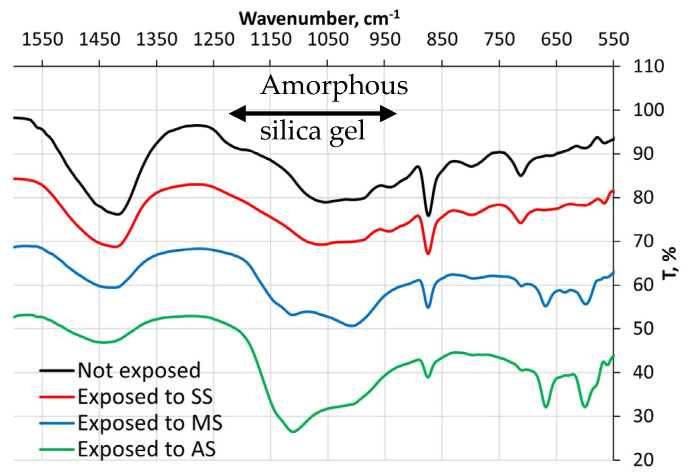
FT-IR spectra of the cement #3 paste samples after 120-day exposure to the sulfate solutions (SS—sodium sulfate, MS—magnesium sulfate, AS—aluminum sulfate).

The Fourier transform infrared (FTIR) spectra obtained from cement #3 paste samples after 120 days of exposure to three types of sulfate solutions are presented in Figure 12. As can be seen from the graph, exposure to magnesium and aluminum sulfates resulted in noticeable changes in the broad absorbance band for silica (in the range of 950–1250 cm^−1^). Specifically, the silica band of the cement #3 paste sample immersed in magnesium sulfate solution appears to have shifted towards the higher wavenumber values (shorter wavelength) and contains two distinguishable peaks. As for aluminum sulfate solution, it seems to affect the silica band, making it shift its peak towards higher values of wavenumbers. The FT-IR spectra belonging to the rest of the pastes are given in Figure S13 (Supplementary Materials).

## 6. Discussion

### 6.1. General Features of the Low-Lime CCS Systems Exposed to Sulfate Solutions

The experimental results collected in the course of this work revealed several characteristics of the CCS systems exposed to sulfate solutions that are quite different from those observed in the OPC systems. First, the CCS matrix does not contain any reactive phases (e.g., calcium hydroxide or aluminates) as it consists mostly of barely soluble (and less reactive) calcium carbonate phases and amorphous silica. As a result, the low-lime CCS matrix is significantly less active towards sulfates compared to the hydrated OPC system. This is reflected in the absence of ettringite deposits as well as in almost complete inertness towards sodium sulfate. In addition, it is necessary to mention that, compared to the hydrated OPC matrix, the CCS matrix is less alkaline, with the highest pH values of the leachate being only ~10. The lower alkalinity of such systems substantially reduces the stability of the products of sulfate attack. Furthermore, unlike in the case of the CSH gel present in OPC systems, the hydrated silica gel present in the CCS matrix does not seem to facilitate the formation of the magnesium silica hydrate (MSH) phase. The formation of the MSH phase in the OPC systems leads to softening of the matrix.

Nevertheless, as it was revealed by the results obtained from various types of the CCS studied in this work, there are some peculiarities and nuances associated with specific types of calcium silicates which should be carefully considered when addressing the sulfate resistance of such systems. That issue is discussed in the next section of this paper.

### 6.2. Effect of the Type of Low-Lime Calcium Silicate on the Sulfate Resistance of the CCS System

As demonstrated by the responses of all CCS pastes exposed to various sulfate solutions used in this work, different carbonated calcium silicates varied in their degree of resistance. While performing a more in-depth analysis of these differences in response, it is important to distinguish between the effects of aluminum sulfate and the effects of the two other sulfates because of the acidic (pH~3–4) nature of the former solution. Specifically, when exposed to aluminum sulfate solution, the matrixes of all cement pastes showed signs of decomposition of calcium carbonate, a significant amount of decalcification of uncarbonated cement grains, a reduction in the amount of leached silica, and the formation of gypsum.

In the case of the other two sulfates, i.e., the sodium and magnesium ones, the level of sulfate resistance depended on the type of calcium silicate present in the binder and the composition of the carbonated phases formed from these silicates. In particular, matrixes resulting from carbonation of wollastonite appeared to be most resistant to both sodium and magnesium sulfates. This is likely because these matrixes contained mostly calcite, the most stable crystalline form of calcium carbonate, as well as relatively highly polymerized amorphous silica with a relatively modest degree of modification by calcium ions [6].

As for the sodium sulfate, it appears that only cements #2 and #3 paste samples participated in the reaction as indicated by the observed formation of gypsum. Due to the presence of less stable calcium carbonate polymorphs (e.g., vaterite), and calcium-modified silica gel with potentially the highest Ca/Si ratio among all the paste samples used in this experiment, the cement #3 paste samples were found to be least resistant to sulfates. The above-mentioned factors were especially critical for pastes in contact with magnesium sulfate. On the other hand, the samples made from cement #4, which contained less hydraulic calcium silicate (larnite), showed higher sulfate resistance when compared to cement #3.

### 6.3. Effect of the Type of the Sulfate Solution on the Sulfate Resistance of the CCS Systems

The dissolution of the components of the main binding matrix, composed of calcium carbonate and amorphous silica, when subjected to a sulfate-rich medium was of primary interest during this study. However, one should not neglect the fact that noncarbonated cement particles might also contribute to calcium and silica leachates. Solubility of a mineral is influenced by several factors, including the activity of an ion, the ionic strength of a solution, the ion-pairing constant (Evangelou, [20]), the pH level of the solution, and the temperature. According to previous reports [21], increasing the ionic strength of the solution (e.g., by adding an inert salt) can facilitate dissolution of otherwise barely soluble minerals.

The ionic strength of sulfate solutions is much higher than that of deionized water (which is considered to be close to zero) and can be estimated by using Equation (1) (Adams [22], Harris [21]).

(1) $$I=\frac{1}{2}\sum C_{i}∙Z_{i}^{2}$$

where *C_i_* is the concentration of an ion *i*, and *Z_i_* is the charge magnitude of an ion *i*.

The calculated values of the ionic strength of the solutions used in the experiments are given in Table 2.

As can be seen from the results, the ionic strength of the sodium sulfate solution was the lowest and that of aluminum sulfate was the highest. These findings imply that certain minerals, e.g., calcium carbonate, may dissolve more easily in MgSO_4_ and Al_2_(SO_4_)_3_ solutions than in the Na_2_SO_4_ solution. An increase in the ionic strength of the solution intensifies the ionic atmosphere around the Ca^2+^ and CO_3_^2−^ ions and results in reduction of their activity coefficients. However, this phenomenon will also depend on the type of polymorph of calcium carbonate, as calcite will be more stable as vaterite or aragonite.

Another factor influencing the solubility of the above-mentioned minerals is the ion-pairing ability of cations and anions present in sulfate solutions, as expressed by the ion-pairing constant (K). According to Evangelou [20], the decrease in the value of the ion-pairing constant increases the stability of the ion–ion pair. The values of the ion-pairing constants for selected ion pairs have been reported by Adams [22]. The equilibrium reactions which can potentially take place in the sulfate soak solutions used in the present study, along with their constants, are given in Table 3.

Comparing the ion-pairing constants, one may conclude that Mg^2+^ and Al^3+^ are more likely to form cation–sulfate anion aqueous pairs than Na^+^. It is also worthwhile to mention that Hasset and Jurinak [23] cited the work published by Akin and Lagerwerff (1965) who suggested that the presence of Mg^2+^ and SO_4_^2−^ in the solution enhances the solubility of CaCO_3_. Furthermore, between Na^+^ and Mg^2+^ ions, the carbonate ion seems to prefer pairing with Mg^2+^ by forming MgCO_3_. This gives rise to the consideration of the possible chemical interaction between calcium carbonate and sulfates.

To evaluate the thermodynamic possibility of room temperature (296 K) chemical reactions between calcium and sulfate ions, changes in the Gibbs free energy of various systems were evaluated, as shown in Equations (2)–(5):

CaCO_3_ + Na_2_SO_4_ + 2H_2_O → Na_2_CO_3_ + CaSO_4_·2H_2_O (2)

∆G ≈ 25 kJ

CaCO_3_ + MgSO_4_ + 2H_2_O → MgCO_3_ + CaSO_4_·2H_2_O (3)

∆G ≈ −36 kJ

3CaCO_3_ + Al_2_(SO_4_)_3_ + 9H_2_O → 2Al(OH)_3_ + 3(CaSO_4_·2H_2_O) + 3CO_2_↑ (4)

∆G ≈ −307.2 kJ

The negative values of the ∆G obtained for the processes shown in Equations (3) and (4) imply that these two reactions are thermodynamically possible. These equations are, however, oversimplified since amorphous silica and/or calcium-modified silica most likely also participate in the reactions. This is indirectly confirmed by observed significant reduction in the amount of leached silica upon exposure to magnesium and aluminum sulfate solutions. In other words, unlike Na^+^ ions, Mg^2+^ and Al^3+^ ions tend to stabilize the silica.

When the reaction involving magnesium sulfate (Equation (3)) has been modified to include silica as an additional reactant (see Equation (5)), the resulting value of ∆G changed from −36 kJ to −230 kJ. This indicates that, thermodynamically, the reaction shown in Equation (5) is more probable than the reaction shown in Equation (3).

CaCO_3_ + MgSO_4_ + SiO_2_ +3H_2_O → MgSiO_3_ + CaSO_4_·2H_2_O + CO_3_^2−^ + 2H^+^ (5)

∆G ≈ −230 kJ

The greater resistance of the CCS pastes to sodium sulfate solution compared to magnesium sulfate solution may be the result of the somewhat less alkaline nature of the latter, as the reduced alkalinity will promote the decalcification of calcium carbonates. In particular, in cases where the carbonated system contains a higher quantity of less crystalline calcium carbonate polymorphs, more calcium ions will be released into solution, thus facilitating the formation of gypsum. As reported earlier [24,25,26], exposure of the OPC system to MgSO_4_ causes disintegration of CSH and formation of gypsum. As such, it is therefore reasonable to assume that in the CCS systems, the Ca-modified hydrated silica gel phase may also contribute calcium ions which, in turn, might react with sulfate derived from MgSO_4_ to form gypsum. Moreover, the negative entropy of the Mg^2+^ ion (≈−138 J/mol·K), as compared to the positive entropy of the Na^+^ ion (≈59 J/mol·K), combined with its smaller ionic radius (0.078 nm for Mg^2+^ vs. 0.098 nm for Na^+^, respectively) will render Mg^2+^ ions more likely to participate in the cation exchange process with calcium ions and to bind with other anionic groups.

During the expansion test reported in [1], the beams samples made from CCS and exposed to sodium sulfate solution did not show any deterioration (expansion values were almost the same as for deionized water). However, powdered paste samples made from cement #3 exposed to sodium sulfate solution were found to contain small (~1 g/100 g of paste vs. ~23 g/100 g of paste in the case of MgSO_4_ solution) amounts of gypsum after 120 days of exposure. This suggests that the carbonated pastes (especially those prepared from cements #2 and #3) can react with Na_2_SO_4_ but do so very slowly. Although initially the well-carbonated calcium silicate system does not contain calcium hydroxide, over time the imbalance between anions and cations (resulting from the release of calcium ions) may lead to reaction between sulfate and calcium ions to form gypsum.

## 7. Conclusions

The results of this study indicate that the resistance of CCS pastes to sulfate attack was influenced by the type of cations associated with a particular sulfate solution and by the composition of the low-lime calcium silicate cements itself. Specifically, the CCS were found to have a higher resistance to Na_2_SO_4_ solution than to MgSO_4_ solution. This is related to the fact that CCS systems do not contain such reactive phases as Ca(OH)_2_ and/or calcium aluminates. Only negligibly small amounts of gypsum were found in the CS pastes exposed to sodium sulfates. On the other hand, CCS were not resistant to the aluminum sulfate solutions, due to their acidic pH values.

CCS containing larger proportions of hydraulic calcium silicates (e.g., cement #3) had lower resistance to MgSO_4_. It is postulated that in this case, the hydrated silica gel might be extensively modified with Ca ions and this modification can therefore lead to a chemical reaction resulting in the formation of gypsum. In addition, the consumption of the magnesium ion and stabilization of silica suggests the interaction between hydrated silica gel and the magnesium cation. This interaction might be due to the cation exchange process taking place between calcium and magnesium ions. This, in turn, underscores the importance, with respect to sulfate resistance, of the calcium ions and silica during the carbonation process as well as the level of pureness of the hydrated silica gel.

The highest resistance to sulfate attack was observed in CCS pastes prepared from carbonated wollastonite, which contained high amounts of calcite and more of highly polymerized silica gel.

## Figures and Tables

**Table 1:** Amounts of carbonates present in the carbonated paste specimens.

Cement Paste	Calcite/Total Amount of Carbonates, g/100 g of the Paste Sample	Presence of the Hump	Hump Temp. Range, °C
#1	41.5	No	-
#2	40.5/42	Yes	567–619
#3	27.2/34	Yes	450–633
#4	32.5/36	Yes	500–632

**Table 2:** Values of ionic strength of the sulfate solutions.

Ion	C*_i_, mol/L	*Z_i_*	*C_i_·Z_i_* ^2^	*I*, mol/L
Sodium sulfate solution				
Na^+^	0.679	1	0.679	1.055
SO_4_^2−^	0.358	2	1.430	
Magnesium sulfate solution				
Mg^2+^	0.370	2	1.480	1.444
SO_4_^2−^	0.352	2	1.408	
Aluminum sulfate solution				
Al^3+^	0.240	3	2.158	1.829
SO_4_^2−^	0.375	2	1.5	

**Table 3:** Ion-pairing constant values.

**Reaction Equation**	**Ion-Pairing Constant (K)** [22]	**Comments**
Ions pairing with sulfate ion		
NaSO_4_^−^ (aq.) ↔ Na^+^ + SO_4_^2−^	2.4 × 10^−1^	Increased likelihood of magnesium and aluminum producing pairs with sulfates.
MgSO_4_^0^ (aq.) ↔ Mg^2+^ + SO_4_^2−^	5.88 × 10^−3^	
AlSO_4_^+^ (aq.) ↔ Al^3+^ + SO_4_^2−^	6.3 × 10^−4^	
Ions pairing with carbonate ion		
NaCO_3_^−^ (aq.) ↔ Na^+^ + CO_3_^2−^	5.35 × 10^−2^	It is more likely that MgCO_3_ (aq.) will be present.
MgCO_3_^0^ (aq.) ↔ Mg^2+^ + CO_3_^2−^	4 × 10^−4^	
For Al^3+^: N/A	N/A	

**Figure A1:**
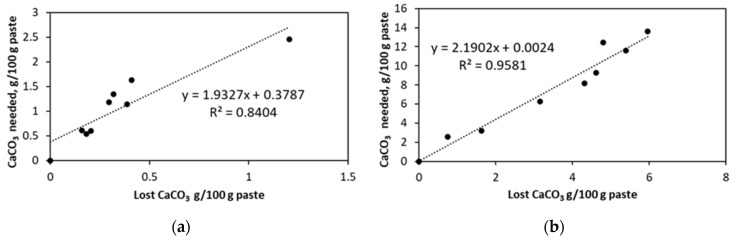
Correlation between lost and needed calcium carbonate amount: (**a**) cement #2 paste, and (**b**) cement #3 paste.

## Data Availability

Not applicable.

## Supplementary Materials

The following supporting information can be downloaded at: https://www.mdpi.com/article/10.3390/ma16124276/s1.

## Appendix A

Equations (3) and (5) (from Section 6) were used to calculate the stoichiometric amounts of calcium carbonate needed to produce gypsum (assuming that the molar ratio of gypsum/calcium carbonate is equal to 1.0). The results of these calculations were compared with the actual amounts of calcium carbonate lost from pastes made from cements #2 and #3 (only these two pastes showed measurable levels of CaCO_3_ loss). The correlations between the calculated (needed) and measured amounts of CaCO_3_ are presented in Figure A1. As seen from the graphs presented in this figure, the formation of gypsum during the sulfate exposure test requires almost twice as much calcium carbonate as the amount of calcium carbonate observed to have been lost during the experiment. This implies that not all of the observed gypsum was formed from the lost calcium carbonate and thus its formation likely involved some other calcium-bearing phases.

Another verification of the correlation of the experimental results with stoichiometric ones was performed for paste samples immersed in aluminum sulfate. As mentioned in Section 5.1, after about the first half day of immersion in aluminum sulfate solution, the amount of sulfate ions was almost depleted together with aluminum ions in the case of all paste samples, which implies that the aluminum and sulfate ions are the rate-controlling species. Therefore, the stoichiometric calculations were performed by implementing the amounts of calcium carbonate in the paste samples and sulfate ion concentration in aluminum sulfate solution determined by TGA and IC, respectively.

The depletion of sulfate ions in early periods of the test can be demonstrated by the stoichiometric calculations based on Equation (4) from Section 6.3.

The concentration of the sulfate ions in the original aluminum sulfate solution was determined by ICP analysis as 0.37 mol/L. Then, if 3 g of paste powder was submerged in 20 mL of solution as per the experiment design, 0.37 mol/L × 0.02 L × 96 g/mol = 0.71 g of sulfate ions becomes available for the reaction. Additionally, according to the estimation from the TGA analysis, 3 g of original paste powder (i.e., before it was exposed to sulfate solution) contained calcium carbonate (calcite) in the range of 27.2–41.5% depending on the type of carbonated cement paste, i.e., 0.82–1.24 g of calcium carbonate. Now, since the molar ratio of the calcium carbonate and sulfate ions in Equation (4) is 1:1, then the following stoichiometric equation is valid:

X     0.71 g CaCO_3_ (aq.) + SO_4_^2−^ + 2H_2_O ↔ CaSO_4_∙2H_2_O + CO_3_^2−^ (A1) 100 g/mol 96 g/mol

Here, X–amount of calcium carbonate in 3 g of paste powder. The value of moles is calculated as υ = X/M [mol], where M is the molar mass of calcium carbonate. Then, depending on the type of the cement paste, ~0.0082–0.0124 moles of calcium carbonate react with ~0.0074 moles of sulfate ions. This proportion clearly shows that the concentration of sulfate ions in the solution is the rate-controlling parameter of the reaction.

These calculations reveal that, theoretically, Χ = (100 × 0.71)/96 ≈ 0.74 g of calcium carbonate is needed to completely consume the available sulfate ions in the solution, which is around 60–90% of the total calcium carbonate amount in original paste powders. The summary of calculated values vs. measured ones (quantified by means of TGA analysis) is presented in Table A1 for all types of paste samples.

**Table A1:** Amount of consumed CaCO_3_ upon exposure to aluminum sulfate solution.

Cement Paste #	Consumed CaCO_3_	
	Calculated	Measured
1	59.4	61.5
2	61	53.4
3	90.1	44.6
4	75.8	60.7

This comparison revealed one interesting point: except for carbonated wollastonite paste (cement paste #1), all other pastes lost smaller amounts of calcium carbonate than those predicted by calculations. This agrees with QXRD data interpretation (see Section 5.2) where the decomposition of uncarbonated calcium silicates by Al_2_(SO_4_)_3_ is claimed. As for the cement paste #1, the lack of drastic difference between calculated and measured values also confirms the QXRD results, showing that the uncarbonated wollastonite particles were decomposed by leftover carbonate ions and that gypsum was mainly formed by the reaction of calcite reacted with aluminum sulfate.
