# Supplementary material for: Resistance of Pastes from Carbonated, Low-Lime Calcium Silica Cements to External Sulfate Attack
Source: Materials (Basel). 2023 Jun 9;16(12):4276. doi: 10.3390/ma16124276 (PMC10302049; doi:10.3390/ma16124276)
Supplement: Supplementary file 1 [file materials-16-04276-s001.zip › materials-2380817-supplementary.pdf]

Table S1. LOI and the amount of total alkalis of the cements

	Cement #1	Cement #2	Cement #3	Cement #4
LOI, %	0.72	1.61	0.71	0.79
Total alkalis, %	0.18	0.45	1.61	0.92

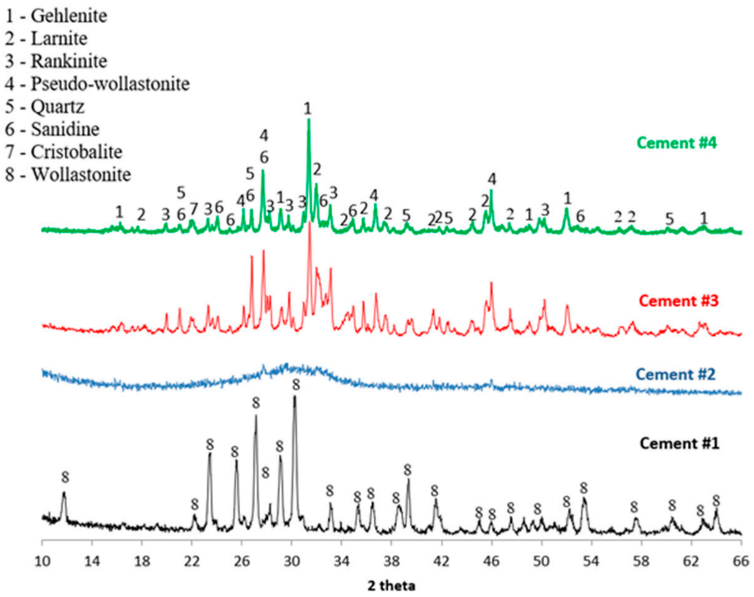

Figure S1. XRD patterns of the cements

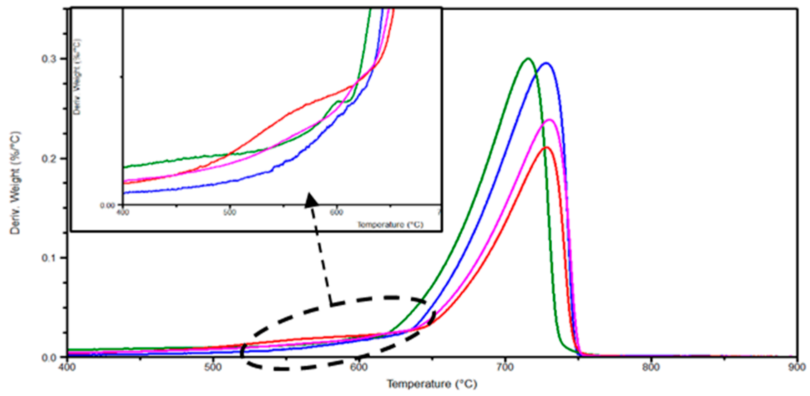

Figure S2. DTGA curves of the carbonated paste samples; left upper corner image contains the magnified view of the hump in the 450-630°C temperature range.

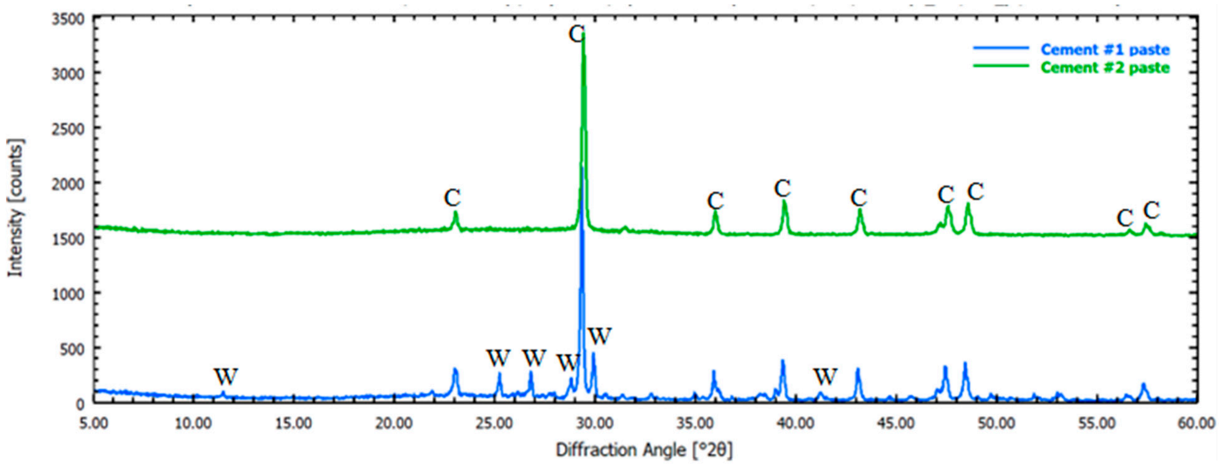

a)

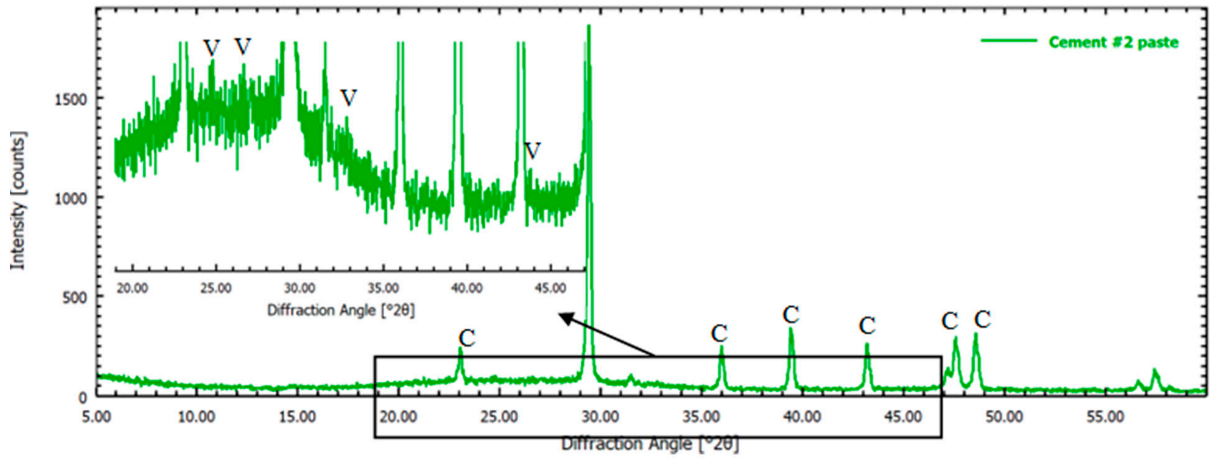

b)

Figure S3. XRD patterns of carbonated cement #1 and #2 pastes: a) both cement pastes with all the main peaks labeled; b) XRD pattern of cement #2 paste with magnified peaks of vaterite (key: C - calcite, V - vaterite, W - wollastonite)

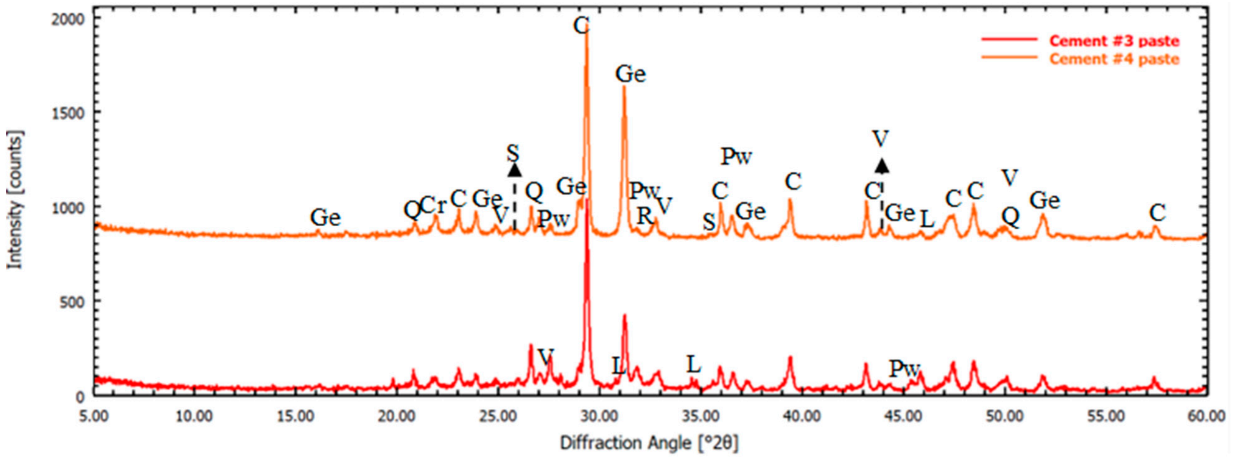

Figure S4. XRD patterns of carbonated cement #3 and #4 pastes (key: C – calcite, Cr – cristobalite, Ge – gehlenite, L – larnite, Pw – pseudo-wollastonite, Q – quartz, R – rankinite, S – sanidine, V – vaterite)

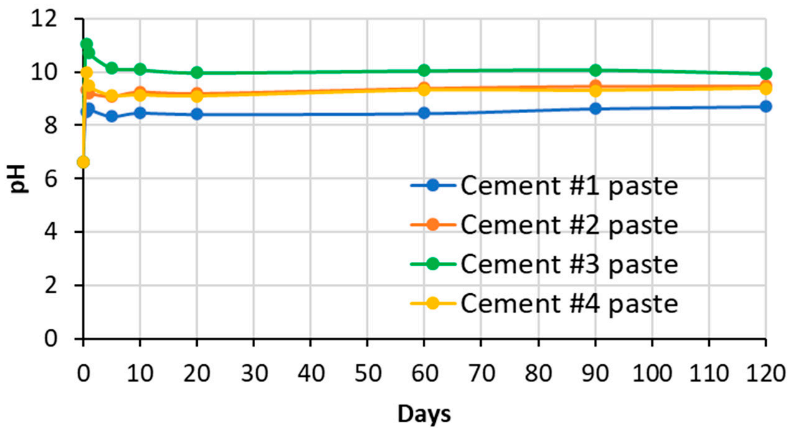
pH
Days
0
2
4
6
8
10
12
0 10 20 30 40 50 60 70 80 90 100 110 120
Cement #1 paste
Cement #2 paste
Cement #3 paste
Cement #4 paste

a)

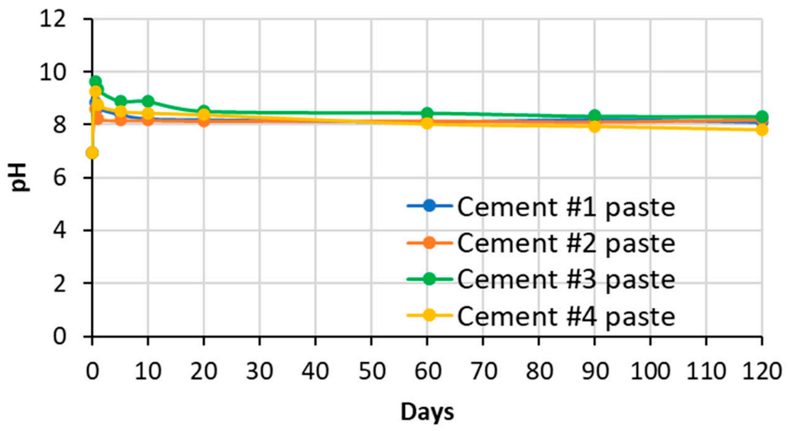
pH
Days
0
2
4
6
8
10
12
0 10 20 30 40 50 60 70 80 90 100 110 120
Cement #1 paste
Cement #2 paste
Cement #3 paste
Cement #4 paste

b)

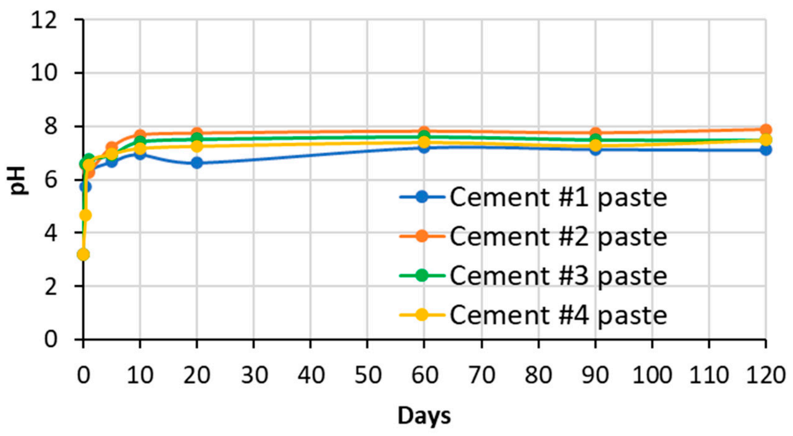
pH
Days
0
2
4
6
8
10
12
0 10 20 30 40 50 60 70 80 90 100 110 120
Cement #1 paste
Cement #2 paste
Cement #3 paste
Cement #4 paste

c)

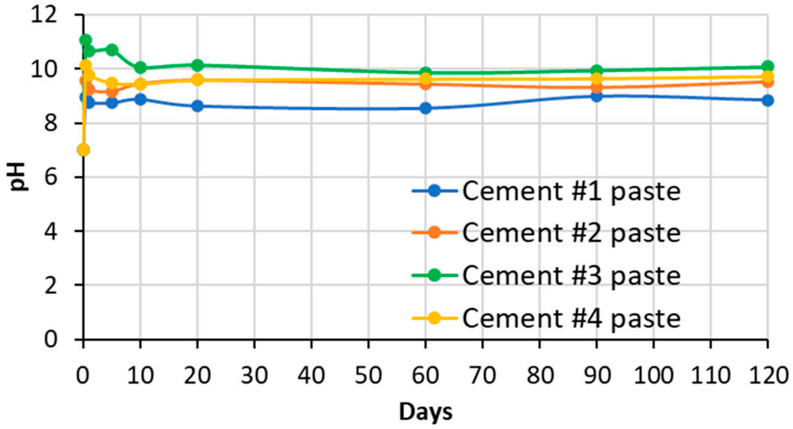

d)

Figure S5. Change of pH values of sulfate solutions a) sodium sulfate, b) magnesium sulfate c) aluminum sulfate solution d)de-ionized water

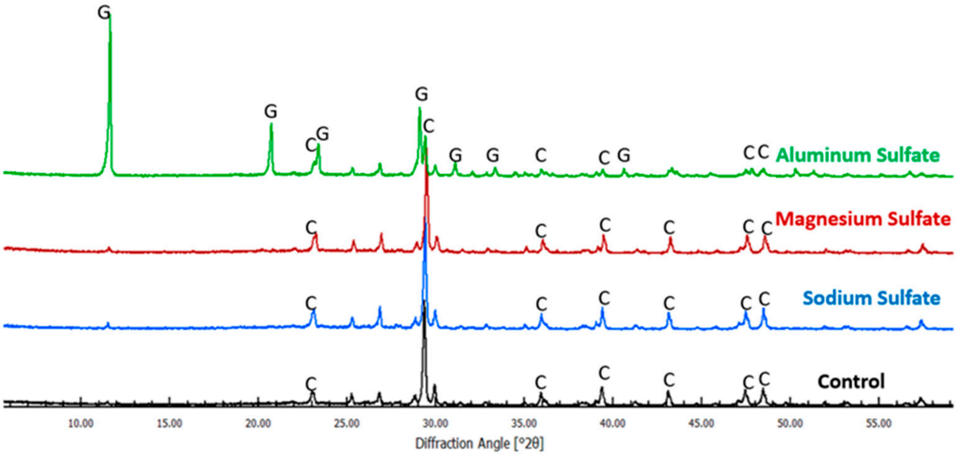

Figure S6. XRD patterns of the cement #1 paste samples after 120-days of exposure to sodium, magnesium and aluminum sulfate solutions (key: C – calcite, G – gypsum)

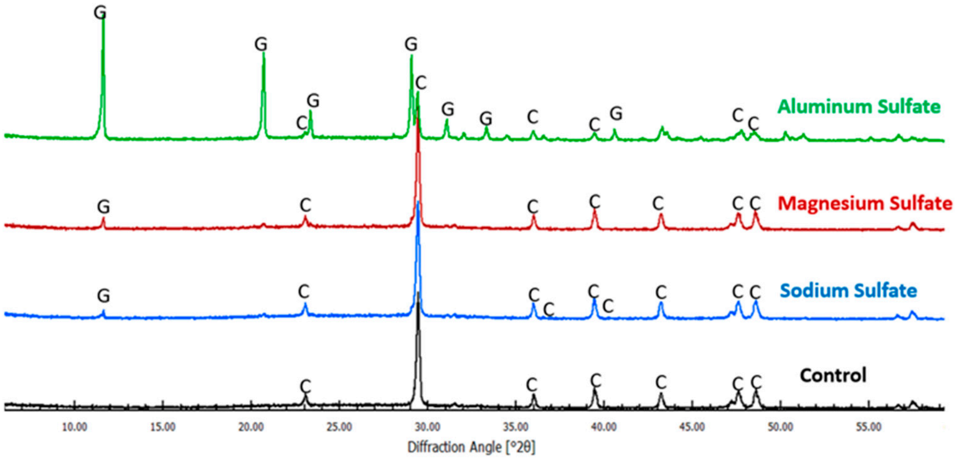

Figure S7. XRD patterns of the cement #2 paste samples after 120-days of exposure to sodium, magnesium and aluminum sulfate solutions (key: C – calcite, G – gypsum)

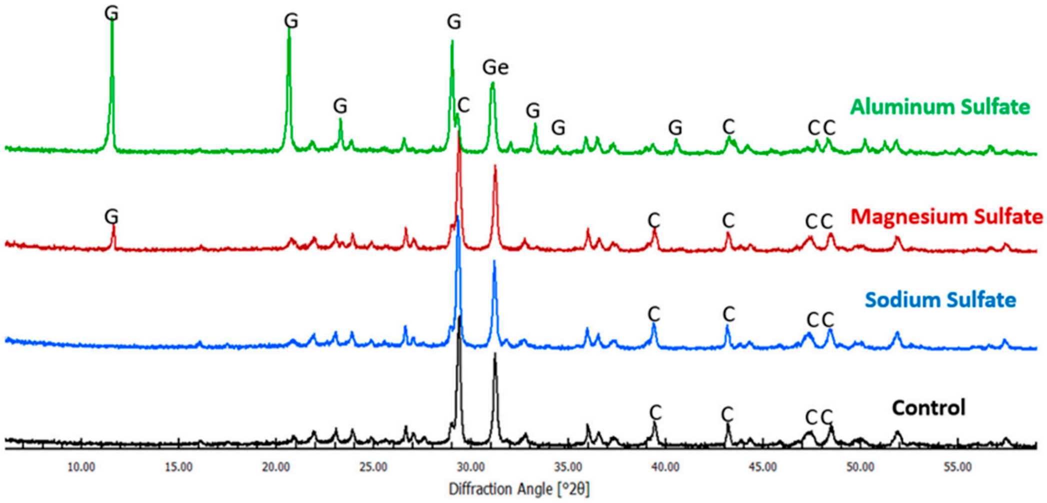

Figure S8. XRD patterns of the cement #4 paste samples after 120-days of exposure to sodium, magnesium and aluminum sulfate solutions (key: C – calcite, G – gypsum)

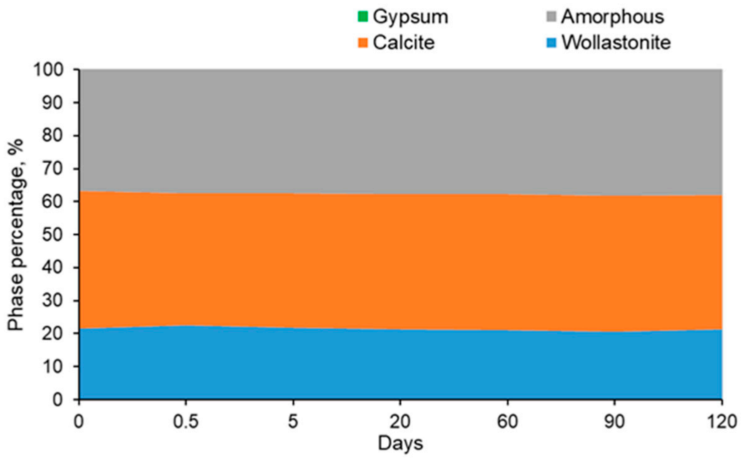

a) After exposer to sodium sulfate solution

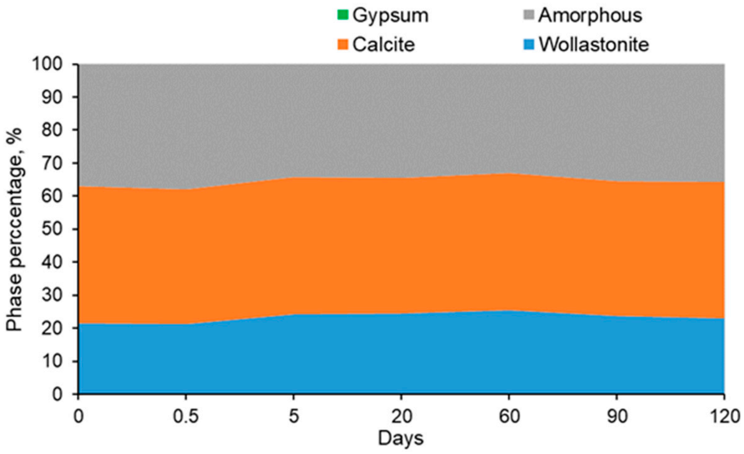

b) After exposer to magnesium sulfate solution

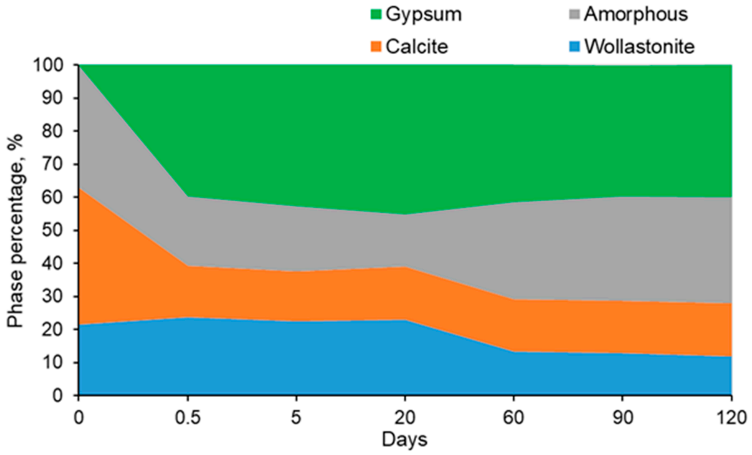

c) After exposer to aluminum sulfate solution

Figure S9. The results of the QXRD analysis of paste samples from cement #1 after sulfate exposure test

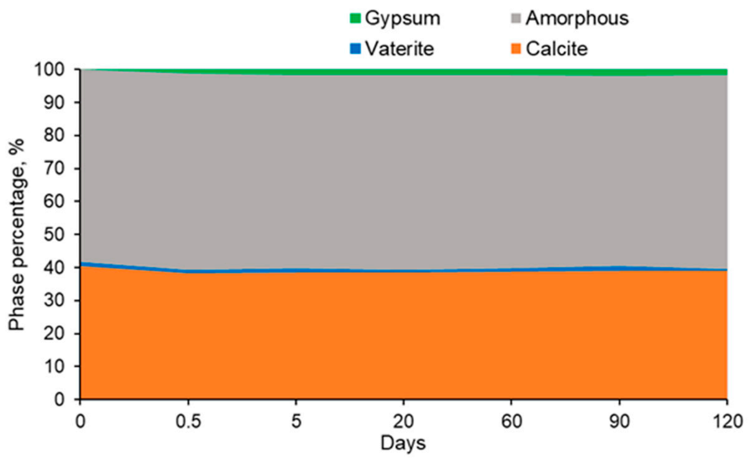

a) After exposer to sodium sulfate solution

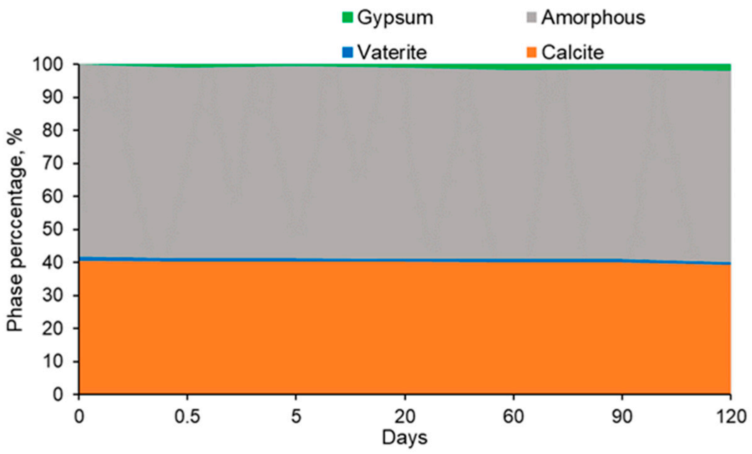

b) After exposer to magnesium sulfate solution

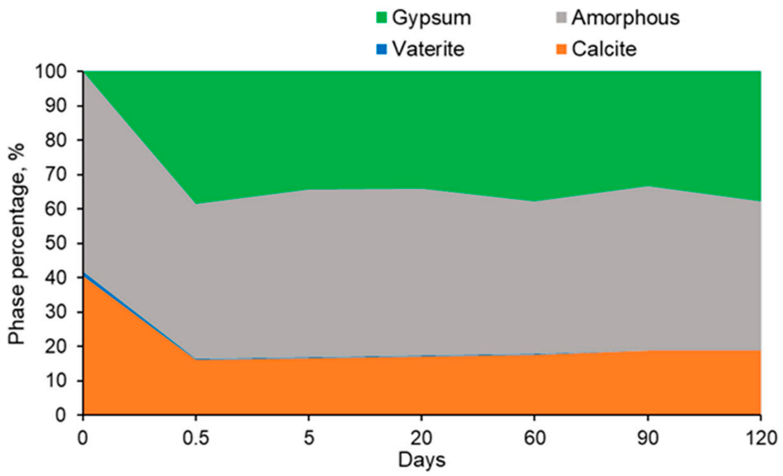

c) After exposer to aluminum sulfate solution

Figure S10. The results of the QXRD analysis of paste samples from cement #2 after sulfate exposure test

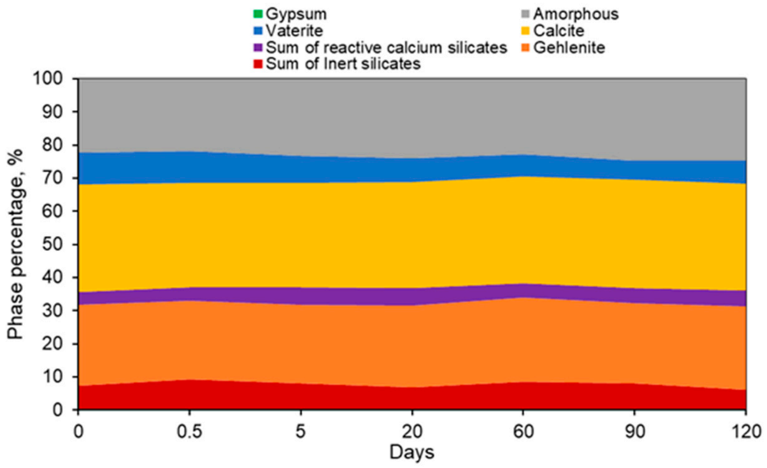

a) After exposer to sodium sulfate solution

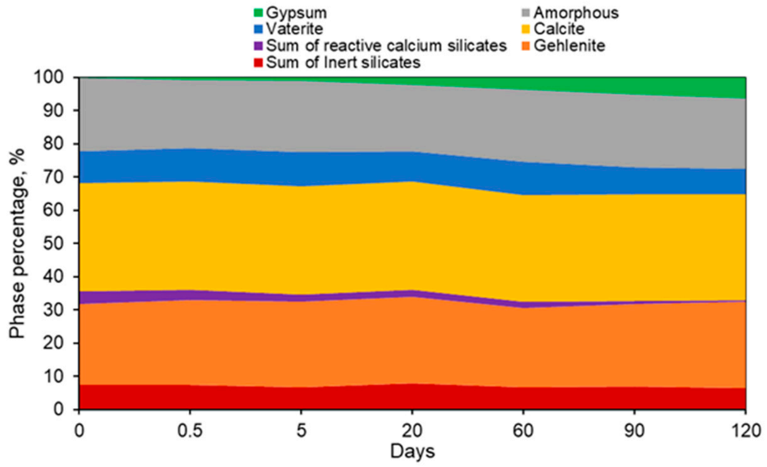

b) After exposer to magnesium sulfate solution

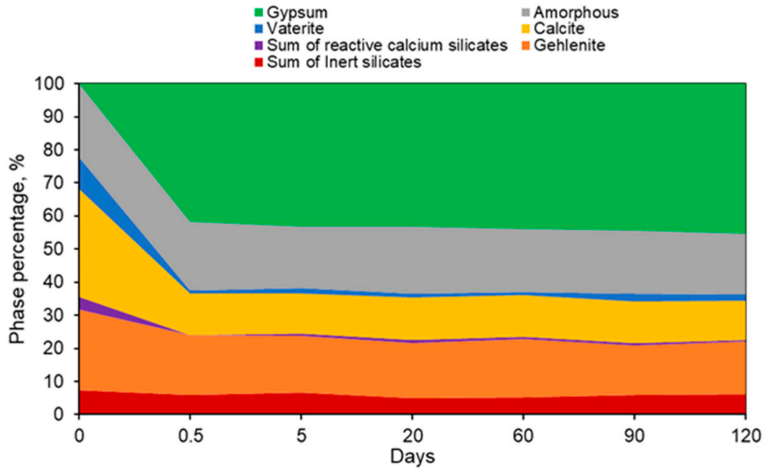

c) After exposer to aluminum sulfate solution

Figure S11. The results of the QXRD analysis of paste samples from cement #4 after sulfate exposure test

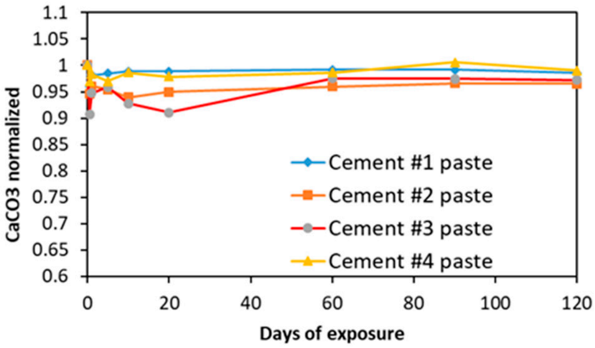

a)

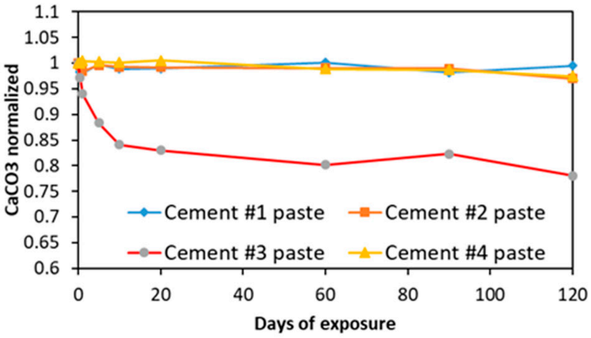

b)

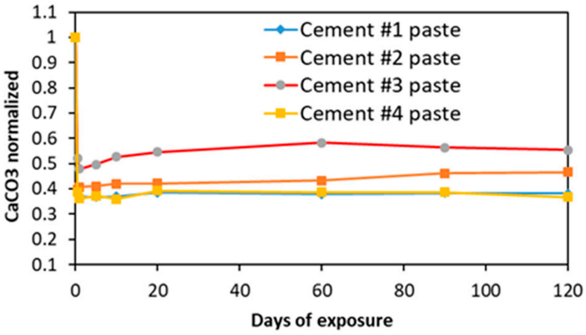

c)

Figure S12. Change of calcium carbonate phase during exposure to a) sodium sulfate, b) magnesium sulfate c) aluminum sulfate solution

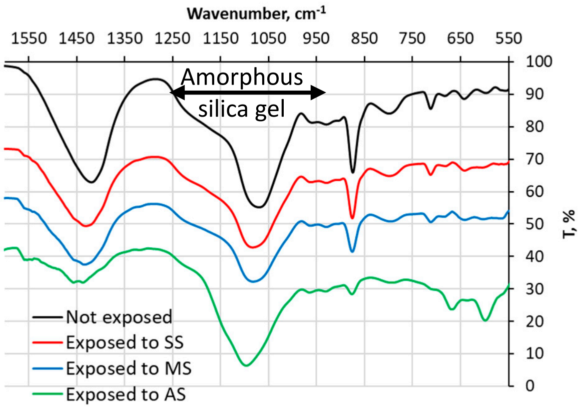

a)

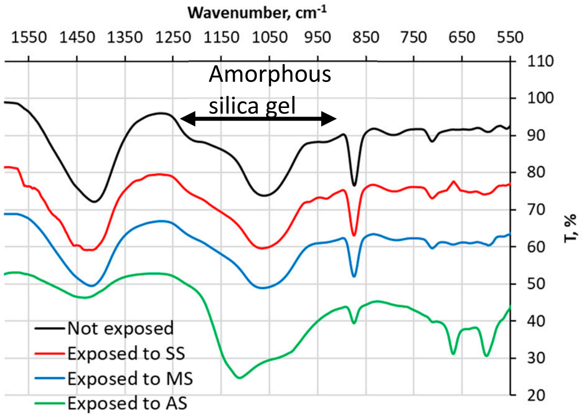

b)

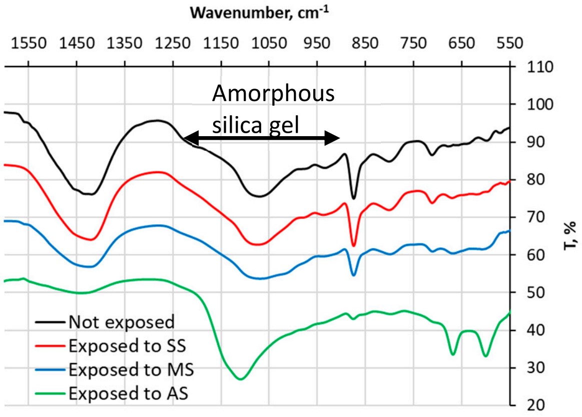

c)

Figure S13. FT-IR spectra of the paste samples after 120-day exposure to the sulfate solutions: (a) – cement #1 paste, (b) – cement #2 and (c) – cement #4 paste samples. Key: SS – sodium sulfate, MS – magnesium sulfate, AS – aluminum sulfate
